# Polymorphisms of NRF2 and NRF2 target genes in urinary bladder cancer patients

**DOI:** 10.1007/s00432-014-1733-0

**Published:** 2014-06-12

**Authors:** Edyta Reszka, Zbigniew Jablonowski, Edyta Wieczorek, Ewa Jablonska, Magdalena Beata Krol, Jolanta Gromadzinska, Adam Grzegorczyk, Marek Sosnowski, Wojciech Wasowicz

**Affiliations:** 1 Department of Toxicology and Carcinogenesis, Nofer Institute of Occupational Medicine, Teresy St. 8, 91-348 Lodz, Poland; 2 I Department of Urology, Medical University, Zeromskiego St. 113, 90-549 Lodz, Poland

**Keywords:** NRF2, Glutathione *S*-transferase, Superoxide dismutase, Genetic polymorphism, Urinary bladder cancer

## Abstract

**Purpose:**

NRF2 transcription factor is involved in modulation of various antioxidant and metabolic genes and, therefore, may modulate anti-carcinogenic potential. Association between polymorphisms of *NRF2* and five NRF2-regulated genes and urinary bladder cancer (BC) risk was analyzed.

**Methods:**

The study group included 244 BC patients, while the control group comprised 365 individuals with no evidence of malignancy. Genotyping of *GSTM1* (deletion), *GSTT1* (deletion), *GSTA1* −69C/T (rs3957357), *GSTP1* Ile105Val (rs1695), *SOD2* Ala16Val (rs4880) and *NRF2* −617C/A (rs6721961) in blood genomic DNA was performed by means of real-time PCR assays. The associations between gene polymorphism and BC risk were computed by logistic regression.

**Results:**

The frequency of *GSTA1, GSTP1, SOD2* and *NRF2* genotypes did not differ in both groups. A significantly higher BC risk was associated with *GSTM1 null* genotype after adjusting to age, sex and smoking habit (OR 1.85, 95 % CI 1.30–2.62; *P* = 0.001). *GSTT1 null* (OR 0.50, 95 % CI 0.31–0.81; *P* = 0.005) and *GSTP1* Val105Val (OR 0.52, 95 % CI 0.27–0.98; *P* = 0.04) genotypes were associated with reduced BC risk separately or in combination (OR 0.24, 95 % CI 0.11–0.51; *P* < 0.0001) (*P* heterogeneity = 0.01). Combined *GSTT1 null* and *SOD2* with at least one 16Val allele among never smokers encompass reduced BC risk (OR 0.14, 95 % CI 0.03–0.63; *P* = 0.01) (*P* heterogeneity = 0.04).

**Conclusions:**

This study supports hypothesis that *GSTM1 null* genotype may be a moderate BC risk factor. The gene–gene and gene–environment interactions associated with combined *GSTP1*/*GSTT1* and combined *GSTT1*/*SOD2* genetic polymorphisms along with cigarette smoking habit may play a significant role in BC risk modulation.

## Introduction

Urinary bladder cancer (BC) is a common disease with high prevalence in the developed countries in comparison with the rest of the world. BC occurs more frequently in men than in women, making it the fourth most common cancer among men and the eighth among women in Europe (GLOBOCAN 2008). Incidence of BC in Poland is slightly lower than the average incidence in Western and Southern Europe, but it has been increasing rapidly. The majority (90–95 %) of BC—transitional cell carcinoma—comprises superficial tumors (70 %), which are usually low-grade and non-muscle-invasive bladder cancer (NMIBC) at the stage Ta/T1, and the second one has the form of muscle-invasive disease (MIBC) at the stages from T2 to T4 (30 %). The overall rate of recurrence for NMIBC ranges from 60 to 70 %, and the overall rate of progression to a higher stage or grade and metastasis from 20 to 30 % (Grotenhuis et al. 2010).

Several well-defined BC risk factors have been identified, including tobacco smoke (encompassing approximately 30–50 % BC risk, similar BC in men and women), aromatic amines, polycyclic aromatic hydrocarbons (PAHs), dietary nitrites and nitrates, chlorinated hydrocarbons, coal, alkylating agents, arsenic, diesel engine exhaust. In addition, BC was one of the first cancers shown to be industrially associated with aniline dye industry (Cohen 1998; IARC 2012; Volanis et al. 2010). BC risk has also been linked with coffee consumption and overall fluid consumption, including chlorinated water. The protective effect on BC risk was attributed to fruit and vegetables consumption and to high selenium status (Brinkman and Zeegers 2008).

Chemically induced urinary bladder carcinogenesis in rodents showed the importance of nuclear factor (erythroid-derived 2)-like 2 (NRF2 or NFE2L2)-regulated signaling pathway (Iida et al. 2004, 2007; Jiang et al. 2009). Moreover, urinary bladder in rats was the most sensitive organ to GST enzyme induction after administration of isothiocyanates, the chemical compounds which contribute well-known NRF2 inductors (Munday and Munday 2002). Various studies have shown that NRF2 signaling pathway is the major mechanism, which controls the expression of target genes with antioxidant response element (ARE) sequence in their promoters. Under basal conditions, transcription factor NRF2 is localized in the cytoplasm and regulated by Kelch-like ECH-associated protein 1 (KEAP1). Alteration of redox balance leads to NRF2 translocation to the nucleus and activation of ARE-containing genes. NRF2-modulated antioxidant that involved in xenobiotic metabolism enzymes may protect against oxidants and electrophilic agents and therefore may contribute to the enhancement of anti-carcinogenic activity (Kensler and Wakabayashi 2010; Maher and Yamamoto 2010). The role of specific NRF2-regulated metabolic and antioxidant genes in urinary bladder tissue has been intensively investigated. It was found that highly metastatic human bladder cells displayed significantly higher mitochondrial superoxide dismutase (SOD2) levels and activities compared with the non-metastatic parental cell line (Hempel et al. 2009). Several studies have shown that glutathione *S*-transferase P1 (GSTP1), GSTM1 and GSTT1 are highly expressed in urinary bladder tissues and showed significantly higher activity and expression of these enzymes in bladder tumors than in normal uroepithelium (Pljesa-Ercegovac et al. 2011; Savic-Radojevic et al. 2007; Simic et al. 2005). The fact that human urinary bladder tumors are characterized by up-regulation of *NRF2* expression in comparison with adjacent non-cancer tissues is also worth attention (Kawakami et al. 2006).

Individual differences in biotransformation of BC carcinogens and in scavenging of reactive oxygen and nitrogen species are quoted as one of the proposed mechanisms in BC etiology. It was observed that cytoprotective genes very often possess ARE sequence and therefore can be modulated by NRF2 transcription factor. For the last two decades, functional polymorphisms of *GSTM1* and *GSTT1* (gene deletion), *GSTP1* Ile105Val (rs1695) affecting gene and enzyme expression, enzyme activity or substrate affinity have been analyzed in relation to BC risk in various ethnic groups. Lack of the enzyme due to gene deletion was observed in case of *GSTM1* (Fryer et al. 1993) and *GSTT1* (Pemble et al. 1994). Minor *GSTP1* 105Val alleles can be associated with lower enzymatic GST activity than major *GSTP1* 105Ile alleles, due to changes in hydrophobic substrate active center (Watson et al. 1998). Functional significance of *GSTA1* −69C/T (rs3957357) polymorphism in promoter region results in differential expression with lower transcriptional activation and lower GST activity of minor *GSTA1* −69T allele than major *GSTA1* −69C allele (Coles et al. 2001). *SOD2* polymorphism (rs4880) is associated with Ala to Val amino acid replacement in codon 16 which results in a conformational change from α-helix to β-sheet in the protein secondary structure and lower mitochondrial import efficiency of the pre-matured SOD2 with *SOD2* 16Val allele than the *SOD2* 16Ala allele (Shimoda-Matsubayashi et al. 1996; Sutton et al. 2003). The functional significance of *NRF2* −617C/A (rs6721961) genetic polymorphism in promoter region is still unraveled. Marzec et al. have found that *NRF2* −617A allele presents significantly lower luciferase activity of promoter construct containing single nucleotide polymorphism relative to the wild type at this locus (*NRF2* −617CC) (Marzec et al. 2007). Recently, Hua et al. (2010) have presented opposite results showing higher luciferase activity of *NRF2* −617A than *NRF2* −617C construct and suggested interaction with triplet repeat polymorphism of *NRF2* (CCG)_4or5_.

Meta- or pooled analyses of GST genetic polymorphism and BC risk, indicated that *GSTM1 null* genotype may be the moderate risk factor (Jiang et al. 2011; Zhang et al. 2011a, b), followed by *GSTP1* 105Val allele (Kellen et al. 2007; Wu et al. 2012). Noteworthy, slight increase in the risk was observed in case of *GSTT1 null* genotype (Gong et al. 2012; Zeng et al. 2010). Unfortunately, only five studies that focus on the role of mitochondrial superoxide dismutase (*SOD2*) (rs4880) genetic polymorphism in BC risk are available (Cengiz et al. 2007; Hung et al. 2004; Kucukgergin et al. 2012; Terry et al. 2005; Vineis et al. 2007) and two studies investigated the role of *GSTA1* (rs3957357) (Komiya et al. 2005; Matic et al. 2013), whereas studies on *NRF2* (rs6721961) genetic polymorphisms in urinary BC risk are still missing. Therefore, the aim of this study was to analyze single and combined polymorphisms of *NRF2* and NRF2 target genes and BC risk, also in relation to potential modifying factors such as age, sex and smoking habit.

## Materials and methods

### Study group

The study group consisted of, in total, 244 BC patients, including 61 females and 183 males, aged 22–92 years (mean age 66.5 years) recruited from the I Department of Urology, Medical University in Lodz, Military Teaching Hospital (central Poland) over the years 2007–2012. The BC patients with previous history of other neoplasms were excluded from the study. All BC patients underwent transurethral resection and they had histopathologically confirmed NMIBC or MIBC at various tumor T stage and degree of G neoplasm. Due to insufficient data regarding T stage of BC patients, only grade G was included in the analyses. The population-based control group of individuals with no evidence of malignancy comprised 365 individuals, including 67 females and 298 males, aged 27–83 (mean age 61.3 years). They were patients of the Primary Health Service at this hospital and volunteers from the Nofer Institute of Occupational Medicine. The Ethics Committee for Scientific Research at Nofer Institute approved the study protocol, and a written informed consent was obtained from each individual before taking part in the study. Venous blood samples were collected into S-Monovette^®^ heparinized test tubes, preserved and stored at −80 °C until DNA isolation. Participants of the study filled in a questionnaire that provided information on demographic characteristics and smoking history with categories of non-, current and ex-smokers. The individuals who declared quitting smoking 5 years and less before the interview were classified as current smokers. Characteristics of the study subjects are presented in Table 1.

**Table 1:** Characteristics of urinary bladder cancer patients and controls

	BC patients, *n* = 244	Controls, *n* = 365	*P* value
Mean age ± SD (min–max)^a^	66.5 ± 10.5 (22–92)	61.3 ± 10.4 (27–83)	<0.0001
Never smokers^b^	65 (27.3 %)	119 (32.8 %)	<0.0001
Current smokers^c^	103 (43.3 %)	196 (54.0 %)	
Ex-smokers	70 (29.4 %)	48 (13.2 %)	
Male	183 (75.0 %)	298 (81.6 %)	0.05
Female	61 (25.0 %)	67 (18.4 %)	
Grade G1^c^	118 (54.1 %)	–	–
Grade G2	61 (28.0 %)		
Grade G3	39 (17.9 %)		

^a^Missing age for eight BC patients and two controls

^b^Missing smoking status for six BC patients and two controls

^c^Missing grade for twenty-six BC patients

### Genotyping

Genomic DNA was isolated from buffy coats using QIAamp DNA Blood Mini Kit (Qiagen), in accordance with the manufacturer’s instructions. Genotyping of *GSTM1* (deletion), *GSTT1* (deletion), *GSTA1* −69C/T (rs3957357), *GSTP1* Ile105Val (rs1695), *SOD2* Ala16Val (rs4880) and *NRF2* −617C/A (rs6721961) in blood genomic DNA was performed by means of real-time PCR assays. Details of the analyzed genetic polymorphisms are presented in Table 2. Primers for end point real-time PCR (*GSTM1* and *GSTT1*) were in accordance with Norskov et al. (2009). HRM (*GSTA1*) assay was designed by the use of Beacon Designer 7.01 (PREMIER Biosoft Int., Palo Alto, CA, USA) based on the GenBank^®^ genetic sequence database with primers: (forward) 5′-TGAAATGTGTGGGAGTGGCTTTT-3′, (reverse) 5′-CGTCCTGGCTCGACAACTG-3′. *GSTM1* and *GSTT1* genotyping was performed using FastStart SYBR Green Master (Roche, Basel, Swizerland) and *GSTA1* using SsoFast™ EvaGreen^®^ Supermix (Bio-Rad Laboratories, Inc., Hercules, CA, USA). Allelic discrimination of *GSTP1*, *SOD2* genotypes was performed by the use of TaqMan^®^ pre-design SNP (single nucleotide polymorphism) assays and TaqMan^®^ custom SNP assay for *NRF2* genetic polymorphism, using TaqMan^®^ Genotyping Master Mix (Life Technologies, Carlsbad, CA, USA). Genotyping was performed using the control DNA samples with known genotypes and a negative control; 10 % of the samples were blindly selected for the re-tests with 100 % concordant results.

**Table 2:** Allelic frequencies in urinary bladder cancer patients and controls

Gene name	dbSNP^a^	Nucleotide/amino acid change	MAF^b^	*χ* ^2c^	*P* value^c^	MAF^b^	*χ* ^2c^	*P* value^c^
			BC patients			Controls		
*GSTM1*	–	Deletion		–			–	
*GSTT1*	–	Deletion		–			–	
*GSTA1*	rs3957357	−69C/T	0.38	0.25	0.62	0.40	1.19	0.26
*GSTP1*	rs1695	562A/G/exon 5, Ile105Val	0.30	0.91	0.34	0.33	0.17	0.68
*SOD2*	rs4880	−9C/T/exon 1, Ala16Val	0.47	2.22	0.14	0.51	4.82	0.03
*NRF2*	rs6721961	−617C/A	0.12	0.17	0.68	0.12	1.41	0.24

^a^dbSNP from http://www.ncbi.nlm.nih.gov/projects/SNP

^b^ *MAF* minor allele frequency

^c^Test of departure from HWE

### Statistical analysis

Arithmetic means with standard deviations and frequencies of the basic characteristics were calculated. The Pearson’s chi-square test was calculated in order to compare the genotype frequencies distribution among the BC patients and controls. A free web-page software based on chi-square test was used to determine any discrepancies of distribution from Hardy-Weinberg equilibrium (HWE) in BC patients and controls (Rodriguez et al. 2009). The associations between single and combined gene polymorphism and BC risk [odds ratio (OR) with 95 % confidence interval (CI)] were computed by the use of logistic regression. The co-dominant model for heterozygous and variant homozygous genotype and dominant model grouping heterozygous with homozygous minor alleles were analyzed. *GSTM1 positive, GSTT1 positive* genotypes and the major homozygotes for *GSTA1, GSTP1, NRF2, SOD2* were set as a reference group. The list of variant genotypes associated with potential risk of BC includes *GSTM1 null, GSTT1 null, GSTA1* −69CT and −69TT, *GSTP1* Val105Val and Ile105Val, *SOD2* Ala16Val and Val16Val, *NRF2* −617CA and −617AA. So as to adjust to the factors that may influence the disease status, age, sex and smoking habit were taken into account. Then, chi-square tests of heterogeneity were carried out. The value of *P* < 0.05 for the group characteristics was considered as the value representing statistical significance. Statistical analyses were performed using STATA11.0 (StataCorp LP, College Station, TX, USA) and GraphPad Prism^®^ version 5.04 (GraphPad Software, Inc., La Jolla, CA, USA).

## Results

The BC patients were significantly older than the individuals from the control group (66.5 vs. 61.3 years, *P* < 0.0001). The investigated groups also differed in terms of sex ratio (*P* = 0.05) and smoking habit (*P* < 0.0001). There were more current smokers in the control group than in the BC patients (54.0 vs. 43.3 %), while ex-smokers were more frequent in the BC group than in the control group (29.4 vs. 13.2 %). The risk of BC among individuals who ever smoked was 1.70 (95 % CI 1.08–2.69; *P* = 0.03), estimated after adjusting for age and gender. Low, G1 grade of urinary bladder tumor was present in 118 of the BC patients, while higher, G2 and G3 grades in 61 and 39 patients, respectively (Table 1).

All genotypes were in HWE in the BC patients and controls (*P* < 0.001) (Table 2). Homozygous deletion of *GSTM1* gene was more frequent in the BC patients than in controls (61.1 vs. 45.2 %, *P* < 0.0001), while homozygous deletion of *GSTT1* gene was more rarely observed in the BC patients than in controls (12.4 vs. 21.1 %, *P* = 0.006). A significantly higher risk of BC was associated with *GSTM1 null* genotype after adjusting to age, sex and smoking habit (OR 1.85, 95 % CI 1.30–2.62; *P* = 0.001). *GSTT1 null* genotype was associated with reduced BC risk (OR 0.50, 95 % CI 0.31–0.81; *P* = 0.005). The frequency of *GSTA1, GSTP1, SOD2* and *NRF2* genotypes did not differ significantly between both groups, but lower prevalence of minor homozygotes was observed in the BC patients in comparison with the controls. *GSTP1* Val105Val genotype was associated with significantly lower BC risk (OR 0.52, 95 % CI 0.27–0.98, *P* = 0.04). That association was not found in the individuals with at least one minor *GSTP1* 105Val allele (OR 0.81, 95 % CI 0.57–1.14; *P* = 0.23) (Table 3).

**Table 3:** Distribution of genotypes and urinary bladder cancer risk

Genotype	BC patients *n* (%)	Controls *n* (%)	*P* value^a^	OR (95 % CI)^b^	*P* value
*GSTM1* positive	95 (38.9)	200 (54.8)	<0.0001	Ref.	
Null	149 (61.1)	165 (45.2)		1.85 (1.30–2.62)	0.001
*GSTT1* positive^c^	212 (87.6)	288 (78.9)	0.006	Ref.	
Null	30 (12.4)	77 (21.1)		0.50 (0.31–0.81)	0.005
*GSTA1 *−69CC^d^	92 (37.9)	137 (37.5)	0.49	Ref.	
−69CT	118 (48.6)	165 (45.2)		0.99 (0.69–1.44)	0.95
−69TT	33 (13.6)	63 (17.3)		0.75 (0.45–1.27)	0.29
-69T	151 (62.1)	228 (62.5)		0.92 (0.65–1.32)	0.66
*GSTP1* Ile105Ile	116 (47.5)	160 (43.8)	0.42	Ref.	
Ile105Val	109 (44.7)	166 (45.5)		0.88 (0.61–1.26)	0.47
Val105Val	19 (7.8)	39 (10.7)		0.52 (0.27–0.98)	0.04
105Val	128 (52.5)	205 (56.2)		0.81 (0.57–1.14)	0.23
*SOD2* Ala16Ala^e^	74 (30.3)	98 (26.9)	0.45	Ref.	
Ala16Val	110 (45.1)	161 (44.2)		0.90 (0.60–1.36)	0.62
Val16Val	60 (24.6)	105 (28.9)		0.79 (0.50–1.25)	0.32
16Val	170 (69.7)	266 (73.1)		0.86 (0.60–1.26)	0.45
*NRF2* −617CC	191 (78.3)	283 (77.5)	0.89	Ref.	
−617CA	49 (20.1)	74 (20.3)		0.97 (0.64–1.49)	0.91
−617AA	4 (1.6)	8 (2.2)		0.49 (0.12–1.94)	0.31
−617A	53 (21.7)	82 (22.5)		0.92 (0.61–1.34)	0.68

^a^Differences in genotypes distribution

^b^Adjusted to age, sex and smoking habit

^c^Genotyping failure for two patients

^d^Genotyping failure for one patient

^e^Genotyping failure for one control

Gene–gene analyses of genotypes with a combination of 15 double genotypes and 18 triple genotypes showed significantly increased BC risk associated with *GSTM1 null* and *GSTA1* −69CT + −69TT genotype (OR 1.56, 95 % CI 1.08–2.26; *P* = 0.02). Significantly reduced BC risk was related to *GSTT1 null* and *SOD2* Ala16Val + Val16Val genotype (OR 0.55, 95 % CI 0.32–0.96; *P* = 0.04) and *GSTP1* Ile105Val + Val105Val and *GSTT1 null* and *SOD2* Ala16Val + Val16Val genotype (OR 0.22, 95 % CI 0.09–0.56; *P* = 0.001). However, the significant impact of gene–gene interaction on BC risk was confirmed only in case of *GSTP1* and *GSTT1* genetic polymorphisms (*P* heterogeneity = 0.01) with reduced BC risk for *GSTT1 null* and *GSTP1* Ile105Val + Val105Val combined genotype (OR 0.24, 95 % CI 0.11–0.51; *P* < 0.0001) (Table 4).

**Table 4:** Combined genotypes associated with urinary bladder cancer risk

Genotype^a^	BC patients *n* (%)	Controls *n* (%)	*P* value	OR (95 % CI)^b^	*P* value
*GSTM1xGSTA1*	150 (61.7)	265 (72.6)	0.005	Ref.	
*GSTM1xGSTA1 variant*	93 (38.3)	100 (27.4)		1.56 (1.08–2.26)	0.02
*GSTP1xGSTT1*	233 (96.3)	320 (87.7)	0.0001	Ref.	
*GSTP1xGSTT1 variant* ^*c*^	9 (3.7)	45 (12.3)		0.24 (0.11–0.51)	<0.0001
*GSTT1xSOD2*	220 (90.9)	311 (85.4)	0.05	Ref.	
*GSTT1xSOD2 variant*	22 (9.1)	53 (14.6)		0.55 (0.32–0.96)	0.04
*GSTP1xGSTT1xSOD2*	226 (97.5)	334 (91.8)	0.003	Ref.	
*GSTP1xGSTT1xSOD2 variant*	6 (2.5)	30 (8.2)		0.22 (0.09–0.56)	0.001

^a^Variant genotypes grouping heterozygous with homozygous minor alleles and *GSTM1 null, GSTT1 null* genotypes; reference genotypes grouping the remaining combination of major, minor and null alleles; the number of subjects may vary from investigated individuals due to *GSTT1* and *GSTA1* genotyping failure

^b^Adjusted to age, sex and smoking habit

^c^ *P* heterogeneity = 0.01

Single and combined genotypes association with BC risk did not vary in groups divided into men and women, and in relation to age and tumor grade G (data not shown). However, a possible association of those genotypes and BC risk stratified by reported smoking status was observed. *GSTM1 null* genotype was associated with BC risk only in current smokers (OR 1.80, 95 % CI 1.09–2.97; *P* = 0.02), but in case of non-smokers and ex-smokers, the estimated risk was similar, although not significant (OR 1.84, 95 % CI 0.93–3.64; *P* = 0.08 and OR 2.04, 95 % CI 0.95–4.36; *P* = 0.08, respectively). BC risk associated with *GSTT1 null* genotype was considerably reduced in non-smokers in comparison with current smokers with *GSTT1 positive* genotype carriers (OR 0.26, 95 % CI 0.09–0.74; *P* = 0.01 vs. OR 0.47, 95 % CI 0.23–0.99; *P* = 0.05). Moreover, several combined variant genotypes among non-smokers were associated with a significantly reduced BC risk: *GSTT1 null* and *GSTP1* Ile105Val + Val105Val (OR 0.07, 95 % CI 0.01–0.57; *P* = 0.01); *GSTP1* Ile105Val + Val105Val and *NRF2* −617CA + −617AA (OR 0.29, 95 % CI 0.08–0.98; *P* = 0.05); *GSTT1 null* and *SOD2* Ala16Val + Val16Val (OR 0.14; 95 % CI 0.03–0.63; *P* = 0.01). The combined *GSTM1 null* and *NRF2* −617CA + −617AA genotypes among current smokers were linked with significantly higher BC risk (OR 2.52, 95 % CI 1.17–5.41; *P* = 0.02). Additionally, a significant impact of smoking habit was confirmed only in case of combined *GSTT1* and *SOD2* genetic polymorphisms in never smokers (*P* heterogeneity = 0.04) (Table 5).

**Table 5:** Single and combined genotypes stratified by smoking status associated with urinary bladder cancer risk

Genotype^a^	Never smokers			Smokers			Ex-smokers		
	BC/Co^b^	OR (95 % CI)^c^	*P* value	BC/Co^b^	OR (95 % CI)^c^	*P* value	BC/Co^b^	OR (95 % CI)^c^	*P* value
*GSTM1*+	22/61	Ref.		44/109	Ref.		29/29	Ref.	
*GSTM1 null*	43/58	1.84 (0.93–3.64)	0.08	59/87	1.80 (1.09–2.97)	0.02	41/19	2.04 (0.95–4.36)	0.08
*GSTT1*+	59/89	Ref.		90/156	Ref.		57/41	Ref.	
*GSTT1 null*	6/30	0.26 (0.09–0.74)	0.01	11/40	0.47 (0.23–0.99)	0.05	13/7	1.23 (0.44–3.42)	0.40
*GSTM1xNRF2*	55/102	Ref.		87/179	Ref.		59/42	Ref.	
*GSTM1xNRF2 variant*	10/17	0.69 (0.27–1.81)	0.46	16/17	2.52 (1.17–5.41)	0.02	11/6	1.30 (0.44–3.88)	0.63
*GSTP1xNRF2*	60/101	Ref.		95/180	Ref.		57/40	Ref.	
*GSTP1xNRF2 variant*	5/18	0.29 (0.08–0.98)	0.05	8/16	0.93 (0.37–2.31)	0.87	13/8	1.10 (0.41–2.94)	0.85
*GSTP1xGSTT1*	64/99	Ref.		97/178	Ref.		66/41	Ref.	
*GSTP1xGSTT1 variant*	1/20	0.07 (0.01–0.57)	0.01	4/18	0.37 (0.12–1.16)	0.09	4/7	0.32 (0.09–1.19)	0.09
*GSTT1xSOD2*	62/98	Ref.		94/168	Ref.		58/43	Ref.	
*GSTT1xSOD2 variant* ^*d*^	3/20	0.14 (0.03–0.63)	0.01	7/28	0.48 (0.20–1.17)	0.11	12/5	1.73 (0.56–5.36)	0.34

^a^Variant genotypes grouping heterozygous with homozygous minor alleles and *GSTM1 null, GSTT1 null* genotypes; reference genotypes grouping the remaining combination of major, minor and null; the number of subjects may vary from investigated individuals due to *GSTT1* and *GSTA1* genotyping failure

^b^Number of urinary bladder patients (BC) versus number of controls (Co)

^c^Adjusted to age and sex

^d^ *P* heterogeneity = 0.04

## Discussion

Specific carcinogens, including occupationally and environmentally derived arylamines and PAHs, require metabolic activation to induce BC. The postulated mechanism of their adverse effect on uroepithelium is based on activities of specific metabolites synthesized in the liver by phase I enzymes and then transported via blood or urine to urinary bladder (Gundert-Remy et al. 2013). However, it was found that local xenobiotics metabolism and also cytoprotective activity may directly influence carcinogenesis process in urinary bladder epithelial cells. The role of NRF2 transcription factor and NRF2-modulated antioxidant and metabolic enzymes against oxidants and electrophilic agents has been widely investigated. Results revealed that human urinary bladder normal and malignant cells displayed high NRF2, SOD2 and GSTs levels (Hempel et al. 2009, Kawakami et al. 2006; Pljesa-Ercegovac et al. 2011; Savic-Radojevic et al. 2007; Simic et al. 2005), which may suggest crucial role in urinary bladder carcinogenesis modulation. For example, the high content of GSTP1 in urothelium may be responsible for the detoxification of benzo[a]pyrene dihydrodiol epoxide (Simic et al. 2009), but at the same time, overexpression of GSTP1, very often observed in urinary bladder tumors, may be linked with drug resistance during chemotherapy (Harbottle et al. 2001). Moreover, genes encoding these metabolic and antioxidant enzymes are known to be polymorphic and they may influence individual’s susceptibility to carcinogens in different ethnic populations (Gong et al. 2012; Jiang et al. 2011; Marzec et al. 2007; Vineis et al. 2007; Wu et al. 2012).

The association between *GSTM1* and *GSTT1* genetic polymorphisms, which are associated with lack of the specific GST isoenzyme and BC risk, has been intensively studied. It is worth to mention that in the present study, we selected those specific genetic polymorphisms, where the link between particular genotype and cancer risk was found to be strictly related to the biological relevance of polymorphism, influencing gene and protein expression or enzymatic activity. Additionally, the reference group for *GSTM1* and *GSTT1* analyses comprised individuals with one and two positive gene copies. This study shows that *GSTM1 null* genotype was associated with significantly increased BC risk (OR 1.85, 95 % CI 1.30–2.62), while *GSTT1 null* genotype with the risk reduction (OR 0.50, 95 % CI 0.31–0.81). Recent two meta-analyses involving 26 (Zhang et al. 2011b) and 33 (Jiang et al. 2011) studies showed that *GSTM1* homozygous deletion was found to slightly influence BC risk in Caucasians and Asians, while in Africans the influence of *GSTM1 null* genotype on cancer risk was not observed. A multi-stage, genome-wide association study including BC cases and controls of European descent confirmed *GSTM1* deletion with *P* = 4 × 10^−11^ and OR 1.47 as a candidate association variant for BC (Rothman et al. 2010).

Although in meta-analysis (Jiang et al. 2011) the effect of *GSTM1 null* genotype on BC risk was increased by smoking habit, the joint effect of *GSTM1 null* genotype was not greater among smokers, never and former smokers in the present study, as well as in New England bladder cancer study (Moore et al. 2011). The marginal association between *GSTT1 null* genotype and BC risk in total ethnic population was found in a meta-analyses including 37 studies (OR 1.12, 95 % CI 1.04–1.21) (Zeng et al. 2010) and in a recent meta-analysis of 50 studies (OR 1.15, 95 % CI 1.04–1.27) (Gong et al. 2012). Opposite to that, in the individuals from central Poland, significantly lower prevalence of homozygous *GSTT1* deletion in the BC patients was observed in comparison with the controls (12.4 % vs. 21.1 %) with OR 0.50 (95 % CI 0.31–0.81). Similarly, a recent study on BC patients living in Dortmund area showed fewer *GSTT1 null* genotypes among cases (17 %) than among controls (20 %) (Ovsiannikov et al. 2012). In the population from central Poland, the protective effect of *GSTT1 null* genotype observed in case of the whole population was definitely more pronounced in non-smokers than in current smokers. Two meta-analyses and New England bladder cancer study showed lack of association between *GSTT1 null* and cancer risk in relation to smoking status (Gong et al. 2012; Moore et al. 2011; Zeng et al. 2010).

Like in the case of *GSTT1* genetic polymorphism, present study also shows protective role of *GSTP1* 105Val variant alleles, associated with defected GST enzymatic activity, in BC risk. Homozygotes with *GSTP1* 105Val allele were more frequent in the controls than in the BC patients (10.7 % vs. 7.8 %) and applied dominant model indicated that *GSTP1* 105Val allele carriers showed non-significantly decreased BC risk (adjusted OR 0.81, 95 % CI 0.57–1.14). Similarly, in the case–control study of BC patients from the USA with 92.9 % of individuals of Caucasian origin in cases, and 97 % in controls, the frequency of *GSTP1* 105Val heterozygotes and homozygotes in controls was higher than in BC cases (Cao et al. 2005). However, a meta-analysis and pooled-analysis indicated that *GSTP1* polymorphism is the modest risk factor for BC with unadjusted summary OR 1.44 (95 % CI 1.17–1.77) for at least one *GSTP1* 105Val allele in case of total ethnic population (Kellen et al. 2007).

Interestingly, our study shows that variant *GSTT1* and *GSTP1* genotypes were associated with reduced cancer risk, which in turn may suggest a complex role of these functional polymorphisms in BC development. We did not observed additional effect of smoking habit on the potential combined *GSTP1* and *GSTT1* polymorphism on BC risk when we stratified patients and controls into never, current and former smokers. In those three subgroups, *GSTT1 null* and *GSTP1* Ile105Val + Val105Val combined genotype was associated with reduced BC risk, as it was observed in total group. It has been found that *GSTT1* gene, under specific carcinogen exposure, plays a critical role in cancer development, due to important contribution of the conjugation reaction catalyzed by GSTT1 to the formation of toxic metabolites, including dihaloalkanes (Monks et al. 1990). Additionally, conformational changes of the GSTP1 105Val alloenzyme may increase *GSTP1* gene expression in human leukocytes and also contribute to more effective detoxification efficacy of PAHs metabolites (Reszka et al. 2011). It should be noted that *GSTP1* Ile105Val (rs1695) polymorphism was also found to be associated with the efficacy of cancer chemotherapy. Lower risk of the disease progression and chemoresistance was found in *GSTP1* 105Val allele carriers (Romero et al. 2012) and also in cancer patients with at least one *GSTP1* 105Ile allele (Zhang et al. 2011a).

In the study of BC patients from central Poland, a significant effect of *GSTA1* −69T allele on BC risk was not observed, but frequency of homozygous carriers of this variant allele was lower in the BC patients than in the controls (13.6 vs. 17.3 %). To compare, in Japanese urothelial cancer patients, also including BC patients, no association between cancer risk and haplotype with minor *GSTA1* −69T allele was found (OR 1.22, 95 % CI 0.87–1.72) (Komiya et al. 2005). Similarly, recent hospital-based case–control study also indicates lack of association between BC risk and at least one *GSTA1* −69T allele (OR 1.34, 95 % CI 0.82–2.20) (Matic et al. 2013). We also observed significantly increased BC risk associated with *GSTM1 null* and *GSTA1* −69CT + −69TT genotype (OR 1.56, 95 % CI 1.08–2.26), which is in agreement with results from the previous study conducted in Serbia, where smoking carriers of those variant genotypes exhibited high risk of BC (OR 2.00, 95 % CI 0.83–4.81) (Matic et al. 2013). Indeed, the combination of variant GST genotypes may be associated with increased oxidative stress and therefore can increase BC risk. Recently, it was observed that oxidative DNA damage measured by urinary 8-hydroxy-2′-deoxyguanosine level was modulated in relation to *GST* genotypes of BC patients. Combined *GSTM1 null* and *GSTA1* −69CT + −69TT genotype connected with low activity was associated with a twofold increase in that oxidative damage (Savic-Radojevic et al. 2013).

In the present study, we did not observe significant impact of *SOD2* Ala16Val polymorphism on BC risk. However, higher frequency of homozygotes with variant *SOD2* 16Val allele was found in the controls (28.9 %) in comparison with the BC patients (24.6 %). Association studies on *SOD2* genetic polymorphism and BC risk showed inconsistent results. In the case–control study of Caucasians from Northern Italy, *SOD2* Val105Val genotype, associated with defective function of SOD2 enzyme, increased BC risk (OR 1.91, 95 % CI 1.20–3.04). Additionally, an effect of minor *SOD2* 16Val allele on BC risk associated with smoking (OR 7.20, 95 % CI 3.23–16.1) or PAHs exposure (OR 3.02, 95 % CI 1.35–6.74) was found (Hung et al. 2004). Higher prevalence of *SOD2* 16Val alleles in control group was observed in the US case–control study (Terry et al. 2005), Caucasians from EPIC cohort (Vineis et al. 2007) and two studies on Turkish individuals (Cengiz et al. 2007; Kucukgergin et al. 2012), which may suggest protective role of *SOD2* 16Val allele in BC risk. Similarly, reduced BC risk associated with *SOD2* 16Val alleles and *GSTT1 null* genotype among non-smokers was also observed in the present study.

The role of transcription factor NRF2 in cancer etiology, its development and treatment is still ambiguous and requires further research. Additionally, NRF2 may affect resistance to common cytotoxic therapies in human cancers (Hu et al. 2010). Moreover, when the activity of NRF2 is too high, it can lead to hyperplasia and increased susceptibility to atherosclerosis. This may serve as an evidence for the hormetic activity of the NRF2 transcription factor (Maher and Yamamoto 2010). The low frequency of *NRF2* −617A variant allele was observed in Caucasian population, including Polish population (12 % in the present study). It was found that minor *NRF2* −617A allele was significantly associated with oxidant-induced acute lung injury among patients of African and European descent with major trauma (Marzec et al. 2007), but it was not associated with gastric carcinogenesis in Japanese patients (Arisawa et al. 2008) or colorectal adenomas in European patients (Tijhuis et al. 2008). In the present study, no association between *NRF2* polymorphism and BC risk was found. However, only four and eight BC individuals with *NRF2* −617AA genotype in BC group and control group, respectively, were found. The present study may indicate association between *NRF2* variants and *GSTM1* and *GSTP1* Ile105Val genetic polymorphisms, however, these interactions were not significant. Interestingly, when the effect of combined polymorphisms was generally uniform across the three strata describing smoking status, *GSTP1* Ile105Val + Val105Val and *NRF2* −617CA + −617AA genotypes significantly reduced BC risk in never smokers (OR 0.29, 95 % CI 0.08–0.98), while the combined *GSTM1 null* and *NRF2* −617CA + −617AA genotypes among current smokers were linked with significantly higher BC risk (OR 2.52, 95 % CI 1.17–5.41).

The results of our study support the hypothesis concerning significant impact of *GSTM1* deletion on BC risk. We also found protective effect of *GSTT1* deletion and *GSTP1* Val105Val genotype (rs1695) on BC risk and lack of such impact of *GSTA1* −69C/T (rs3957357), *SOD2* Ala16Val (rs4880) and *NRF2* −617C/A (rs6721961). Additionally, gene–gene and gene–environment interactions modulating BC risk were observed for *GSTT1* and *GSTP1* genotype, *GSTT1* and *SOD2* genotype and smoking habit. Taking into account several limitations of the study, including small sample size and selection bias of individuals from general population, these conclusions should be regarded with caution. In addition, the study was underpowered in terms of the assessment of moderate and small effects of minor alleles and of gene–gene and gene–environment interactions. However, the present study achieved power of at least 80 % to observe associations of the magnitude of OR 1.8 with *GSTM1 null* genotype frequency of about 50 % and OR 0.5 with *GSTT1 null* genotype frequency of about 20 %. Further studies taking into account various confounding variables, such as adequate number of individuals, study design and control selection, may explain still ambiguous results of investigations undertaken to clarify the correlation between *NRF2* and NRF2-target genes polymorphisms and BC risk.
